# Highlights from Heart Rhythm 2018: Late-breaking Clinical Trial Considerations

**DOI:** 10.19102/icrm.2018.090909

**Published:** 2018-09-15

**Authors:** Brian Olshansky

**Affiliations:** ^1^Department of Cardiology, University of Iowa, Iowa City, IA, USA

**Keywords:** Ablation, atrial fibrillation, heart block, syncope

## The CABANA trial

The Catheter Ablation versus Antiarrhythmic Drug Therapy for Atrial Fibrillation (AF) (CABANA) trial was one of the most watched and scrutinized trials presented^1^ at this year’s Heart Rhythm Society Scientific Sessions. This prospective randomized trial of ablation versus medical therapy for AF was supported by the National Institutes of Health (Bethesda, MD, USA), Biosense Webster (Diamond Bar, CA, USA), the St. Jude Foundation and Corporation (St. Paul, MN, USA), and Boston Scientific Corporation (Natick, MA, USA) and included patients who were considered candidates for ablation or medical therapy for the treatment of AF. Notably, the inclusion criteria were not expounded upon in detail during the presentation; what we do know is that patients who were enrolled had new-onset (less common) or “undertreated” paroxysmal, persistent, or longstanding persistent AF and warranted the use of therapy of some kind. Key inclusion criteria included (1) an age of either 65 years or older or that younger than 65 years with one or more cerebrovascular accidents/cardiovascular risk factors (eg, hypertension, diabetes, congestive heart failure) and (2) eligibility for ablation and two or more rhythm or rate control drugs. Exclusion criteria, although not discussed in the presentation, included a number of items such as the presence of lone AF in the absence of risk factors for stroke in patients younger than 65 years, those who in the opinion of the managing clinician should not yet receive any therapy for AF; those who had experienced other cardiac events in the preceding three months such as myocardial infarction, percutaneous coronary intervention, or valve or bypass surgery; those with hypertrophic obstructive cardiomyopathy; those with renal failure requiring dialysis; those with class IV angina or class IV chronic heart failure (including past or planned heart transplantation); and those with other arrhythmias mandating antiarrhythmic drug therapy (eg, ventricular tachycardia or ventricular fibrillation).^2^

Patients in the CABANA trial were randomized 1:1 to “primary” ablation (n = 1,108) such as pulmonary vein isolation/wide-area circumferential catheter ablation and ancillary ablation including linear lesions and targeting of complex fractionated electrograms; additionally, these patients were given anticoagulants. This group was compared to a drug therapy arm (n = 1,096) who were given anticoagulants and rate and/or rhythm control medications; however, importantly, this group was not carefully controlled regarding the type of drug therapy being taken (rate versus rhythm) or endpoint (rhythm control or rate control). Moreover, this group may have included individuals who were already treated with a similar therapy before enrollment.

The idea of the CABANA trial was to compare “state-of-the-art” (not otherwise specified) drug therapy to catheter ablation, with the primary combined endpoint of all-cause mortality, disabling stroke, serious bleeding, or cardiac arrest (which was not the original primary endpoint). There were secondary endpoints, but none that specifically considered symptoms or AF burden.

Importantly, of those randomized to ablation, 90.8% had ablation and 90.4% completed follow-up. Thus, 9.2% were crossed over to drug therapy in this arm and nearly 10% were lost to follow-up. In the drug therapy arm, 87.2% underwent a rhythm control drug approach and a total of 27.5% were crossed over at some point during follow-up to ablation. Twelve percent were lost to follow-up in this arm. Thirty-seven percent were female and only about 14% were 75 years or older. Approximately 40% were hospitalized in the past for AF, and AF had started a median of 1.1 years before enrollment in the CABANA trial. Various forms of AF were considered—specifically, paroxysmal, persistent, and longstanding persistent.

In terms of the primary endpoint outcome by intention-to-treat analysis, there were no differences in outcomes. Similarly, there was no difference in mortality between the study arms. When considering the components of the primary and secondary outcomes, the only difference was in the combined endpoint of death or cardiovascular hospitalization (51.7% versus 58.1%), but there was no difference in mortality. Thus, the entire and rather small difference was in regard to cardiovascular hospitalization.

Regarding time to first recurrence of AF, there was a significant difference (postblanking) between the two groups, with the drug arm having no blanking period and the ablation arm having one. Data on AF burden were not presented, nor were data on any time-dependent relationship between AF recurrence based upon the use of drug or ablation therapy and outcomes offered.

The most controversial aspect of this presentation was the consideration of post-hoc “on-treatment” results showing a significant difference in the primary outcome, all-cause mortality, and death or cardiovascular hospitalization. Adverse events were low in both groups, suggesting that present medical treatment options are associated with good and perhaps better than expected outcomes.

The first conclusion presented was that ablation did not produce a significant reduction in the primary endpoint in all-cause mortality, but the results were affected by crossovers in both directions and lower-than-expected event rates.

Overall, these results were perplexing and probably will not change how we presently ablate AF in our patients. Although the study based on the combined (revised) primary endpoint was negative, the data left some room for interpretation. Specific weaknesses of the trial were (1) long time for enrollment; (2) selective enrollment, which is hard to generalize to the overall AF population; (3) lack of an adequate matched control; and (4) expectations of patients for a cure with ablation versus a lack of expectation with continuing an old or alternative antiarrhythmic drug therapy. The impact of this is unknown, but the nocebo effect of drug therapy versus the hope of an ablation cannot be underestimated. This specifically applies to the crossover patients in the drug therapy arm who ended up undergoing an ablation. These patients were not adequately randomized. Additionally, there was (5) a lack of detailed guidance on medical therapy; (6) a high crossover rate that was in part expected but difficult, if not impossible, to interpret and, while hypothesis-generating, does not provide evidence one way or another for ablation based on the primary or any endpoint; and (7) a lack of correlation of AF recurrence to outcome measures such as mortality. It is also important to consider that (8) endpoints, including mortality, that are rarely if ever considered a reason to ablate patients with AF, were used; (9) there were low event rates in both groups, perhaps due to the medical therapy given in both groups or the overestimation of mortality with AF; (10) there was difficulty in the interpretation of time-to-first recurrence of AF or importance of AF recurrence; (11) there was a high number of patients who were lost to follow-up (what if all those in the ablation or drug therapy arm who were lost to follow-up died, for example?); and (12) there may be difficulty in generalizing these data to an unenrolled population.

As these data and related subanalyses become available in publications, further understanding of these data will become apparent, but, at this point, these results are not totally unexpected and do not change the practice of medical therapy or catheter ablation in most patients with AF.

## Permanent pacemaker in patients with syncope and bifascicular block

In a separate late-breaking clinical trial at Heart Rhythm Society 2018, Sheldon et al.^3^ assessed the value of permanent pacemaker implantation for patients who have bifascicular block and who had at least one syncopal spell in the year prior to implant. In this multinational trial involving patients older than 50 years with normal ventricular function with at least one syncopal spell in the prior year and right bundle branch block and left anterior hemiblock (n = 75) or left bundle branch block (n = 40), 56 subjects received a permanent pacemaker and 59 subjects received an implantable cardiac monitor, with the primary composite outcome measure being syncope, death, symptomatic bradycardia, asymptomatic actionable bradycardia, and device complications. Over a median follow-up period of 30 months and with 18% of participants exiting the study early, 63% in the permanent pacemaker arm and 22% of those in the implantable cardiac monitor arm were found to be free of the combined endpoint. Thirty of the 59 (51%) patients in the implantable cardiac monitor arm crossed over to a permanent pacemaker arm (but the remainder did not).

While empiric implantation of a pacemaker seems reasonable based on these data, in this population of patients with syncope purportedly due to bradycardia caused by conduction system disease, five patients who received pacemakers had complications. Also, the urgency to have a pacemaker implant was not clearly present, since the proportion of syncope was similar in both groups and the reason for the crossover in the cardiac monitor arm was generally just mildly symptomatic bradycardia. Thus, while permanent pacing may be the preferred strategy in these patients, no major harm was seen with an initial strategy of an implantable loop recorder first, and perhaps some patients may be spared an unnecessary pacemaker placement and subsequent complications if empiric pacemaker implantation was not the initial strategy. Furthermore, a pacemaker may not prevent all episodes of syncope in this population.
